# New Method of Percutaneous Revascularization Treating Severe and Hard-to-Treat Chronic Limb-Threatening

**DOI:** 10.12669/pjms.42.4.14356

**Published:** 2026-04

**Authors:** Emced Khalil

**Affiliations:** 1 Dr. Emced Khalil Associate Professor, Trabzon Ahi Evren Cardiothoracic Surgery, Training and Education Hospital, Trabzon, Turkey

**Keywords:** Amputation-free survival, Chronic limb-threatening ischemia, Endovascular revascularization, Major adverse limb events, Peripheral artery disease, Quality of life

## Abstract

**Background & Objective:**

Chronic limb-threatening ischemia (CLTI) represents the most severe form of peripheral artery disease and is associated with high risks of limb loss and mortality. Contemporary management increasingly favors endovascular revascularization; however, optimal outcomes depend on structured multidisciplinary care in addition to technical success. This study aimed to evaluate clinical and hemodynamic outcomes of endovascular revascularization performed within a multidisciplinary limb salvage program in patients with advanced CLTI, with a focus on amputation-free survival (AFS) and limb preservation.

**Methodology:**

This retrospective single center study included 79 consecutive patients with advanced CLTI who underwent endovascular revascularization between January 2019 and December 2023. All patients received standardized guideline-directed medical therapy and multidisciplinary postoperative care. Hemodynamic improvement was assessed by pre- and post-procedural ankle-brachial index (ABI). AFS was evaluated using Kaplan-Meier analysis at 12 months. Univariable Cox regression analysis was performed to explore predictors of adverse AFS events.

**Results:**

Mean ABI increased significantly from 0.61 ± 0.20 preoperatively to 0.89 ± 0.23 postoperatively (paired t test, p < 0.001). Kaplan-Meier analysis demonstrated a 12-month amputation-free survival rate of 89.2% (95% CI, 82.1-96.3). Primary and secondary patency rates were 67.1% and 27.8%, respectively. Major amputation occurred in 3.8% of patients, while overall mortality was 1.3%. In univariable analysis, diabetes mellitus and total arterial occlusion were significantly associated with reduced amputation-free survival.

**Conclusion:**

Endovascular revascularization integrated within a multidisciplinary limb salvage program is a safe and effective strategy for patients with advanced CLTI. This approach achieves significant hemodynamic improvement, high amputation-free survival, and low rates of major limb loss despite complex anatomy and severe disease burden.

## INTRODUCTION

Chronic limb-threatening ischemia (CLTI) represents the most advanced and debilitating manifestation of peripheral artery disease (PAD), characterized by ischemic rest pain, non-healing ulcers, or gangrene. Patients with CLTI face a substantial risk of limb loss, impaired functional capacity, reduced quality of life, and increased mortality, making timely and effective revascularization essential for limb preservation.^1^,^2^ Contemporary international guidelines emphasize prompt restoration of perfusion as the cornerstone of CLTI management, particularly in patients with advanced ischemic and anatomical disease severity.^1^ Over the past two decades, major technological advances in endovascular devices, guidewire systems, and imaging techniques have transformed endovascular revascularization into a widely accepted first-line strategy for many patients with CLTI.^3^-^5^

Randomized trials and contemporary registries have demonstrated that endovascular-first approaches can achieve limb-salvage outcomes comparable to surgical bypass, while offering advantages in perioperative safety and recovery, especially in high-risk patient populations.^2^,^3^ Nevertheless, successful limb salvage in CLTI extends beyond technical revascularization alone. Increasing evidence highlights the critical importance of structured multidisciplinary care, integrating vascular surgeons, endovascular specialists, diabetologists, wound-care teams, infectious disease specialists, and rehabilitation services.^6^-^8^ Such collaborative models have been shown to improve wound healing, reduce infection-related complications, limit major adverse limb events (MALE), and enhance amputation-free survival (AFS), particularly in patients with advanced WIfI stages.^7^,^8^ Despite these advances, real-world data evaluating outcomes of endovascular revascularization performed within dedicated multidisciplinary limb-salvage programs remain limited, especially among patients with complex anatomy, extensive occlusive disease, and high clinical risk profiles as defined by WIfI and TASC II classifications. Moreover, long-term limb preservation in CLTI depends not only on vessel patency but also on optimal medical therapy, antithrombotic management, and coordinated postprocedural follow-up.^8^

Therefore, the present study aimed to evaluate the clinical, hemodynamic, and limb-related outcomes of endovascular revascularization performed within a multidisciplinary limb-salvage program in patients with advanced CLTI, with a particular focus on amputation-free survival, patency, limb preservation, and perfusion improvement.

## METHODOLOGY

This retrospective, single-center observational study included consecutive patients diagnosed with chronic limb-threatening ischemia (CLTI) who underwent endovascular revascularization between January 2019 and December 2023 at a tertiary cardiovascular surgery center. CLTI was defined by the presence of ischemic rest pain, non-healing ischemic ulceration, or gangrene, in accordance with contemporary guideline definitions.^1^,^5^

Patients aged ≥18 years with advanced CLTI were eligible for inclusion if they met both clinical and hemodynamic criteria. Clinical severity was assessed using Rutherford and WIfI classifications, and anatomical complexity was evaluated according to the TASC II system.

### Ethical considerations:

The study was conducted in accordance with the Declaration of Helsinki and approved by the institutional ethics committee (Approval No: 2021/203; Date: September 9, 2021). Written informed consent was obtained from all patients or their legal representatives prior to inclusion.

### Inclusion criteria:

- Age ≥18 years.
- CLTI with rest pain, ulcer, or gangrene.
- Predominantly advanced disease (Rutherford class ≥4; WIfI stage 3-4).
- Hemodynamic confirmation of ischemia by reduced ankle-brachial index (ABI).
- Imaging-confirmed atherosclerotic lower-extremity arterial disease.

### Exclusion criteria:

- Acute limb ischemia.
- Non-atherosclerotic arterial disease.
- Prior major (above-ankle) amputation of the target limb.
- Severe systemic infection precluding endovascular intervention.
- Incomplete clinical or follow-up data.

### Preprocedural Assessment and Medical Management:

All patients underwent standardized preprocedural evaluation including detailed clinical examination, ABI measurement, and vascular imaging. Guideline-directed medical therapy was initiated or optimized in all patients and included antiplatelet therapy, high-intensity statin therapy, strict control of cardiovascular risk factors (diabetes mellitus, hypertension, and dyslipidemia), and targeted infection management when required. Wound assessment and debridement were performed in collaboration with wound-care specialists as part of the multidisciplinary limb-salvage program.

### Endovascular Revascularization Procedure:

Endovascular interventions were performed by experienced operators using standard percutaneous techniques. Lesion morphology and anatomical complexity were classified according to the TASC II criteria. Revascularization strategies included conventional balloon angioplasty and drug-coated balloon angioplasty, selected at the operator’s discretion based on lesion characteristics. Stent implantation was reserved for cases with flow-limiting dissection, significant elastic recoil, or residual stenosis following angioplasty. Technical success was defined as <30% residual stenosis with restoration of inline flow to the distal runoff vessels.

### Postprocedural Care and Follow-up:

Postprocedural management consisted of continuation of antiplatelet therapy, optimization of medical treatment, structured wound-care follow-up, and rehabilitation when indicated. Patients were followed at 1, 3, 6, and 12 months after the index procedure with clinical assessment, ABI measurement, duplex ultrasonography, and wound evaluation. Limb outcomes, reinterventions, amputations, and survival status were prospectively recorded during follow-up visits.

### Study Endpoints and Definitions:

The primary endpoint was amputation-free survival (AFS), defined as freedom from major (above-ankle) amputation or all-cause mortality, irrespective of the underlying cause (cardiovascular or non-cardiovascular). Secondary endpoints included changes in ABI, primary and secondary patency, limb salvage, and rates of major and minor amputations.

### Statistical analysis:

Continuous variables are presented as mean ± standard deviation (SD), and categorical variables as counts and percentages. Normality was assessed using the Shapiro-Wilk test. Pre- and postprocedural ABI values were compared using a paired-samples *t* test or Wilcoxon signed-rank test, as appropriate.

Amputation-free survival was estimated using the Kaplan-Meier method, and the 12-month AFS rate was reported with 95% confidence intervals (CIs). Patients without events were censored at their last follow-up visit. Univariable Cox proportional hazards regression analysis was performed to explore the association between baseline variables-including diabetes mellitus, chronic kidney disease, and total arterial occlusion-and AFS. Hazard ratios (HRs) with 95% CIs were calculated. A two-sided p value <0.05 was considered statistically significant. Statistical analyses were performed using SPSS software (IBM Corp., Armonk, NY).

## RESULTS

A total of 79 patients with advanced chronic limb-threatening ischemia (CLTI) were included in the study. The mean age was 65.1 ± 13.1 years, and the majority of patients were male (93.7%). Diabetes mellitus was present in 46.8% of patients, while 41.8% had hyperlipidemia and 29.1% had concomitant coronary artery disease. Chronic kidney disease was observed in 5.1% of the cohort. Most patients presented with severe ischemia, with 78.5% classified as Rutherford class ≥4 and 97.5% as WIfI stage 3-4. Anatomically complex disease was common, with TASC II C-D lesions identified in 78.5% of patients and total arterial occlusion present in 51.9%. Target lesions were predominantly located in the femoropopliteal segment (69.6%), while infrapopliteal involvement was observed in 30.4% of cases (Table-I).

**Table-I:** Baseline Demographic and Clinical Characteristics.

Variable	n / Mean ± SD	%
Age (years)	65.05 ± 13.07	-
Male sex	74	93.7
Smoking	31	39.2
Diabetes mellitus	37	46.8
Hyperlipidemia	33	41.8
Coronary artery disease	23	29.1
Chronic kidney disease	4	5.1
Rutherford class ≥4	62	78.5
WIfI stage 3-4	77	97.5
TASC II C-D lesions	62	78.5
Total occlusion	41	51.9
Femoropopliteal segment	55	69.6
Infrapopliteal arteries	24	30.4

***Abbreviations:*** WIfI, wound-ischemia-foot infection; TASC, TransAtlantic Inter-Society Consensus.

### Procedural Characteristics:

**Fig.1:**
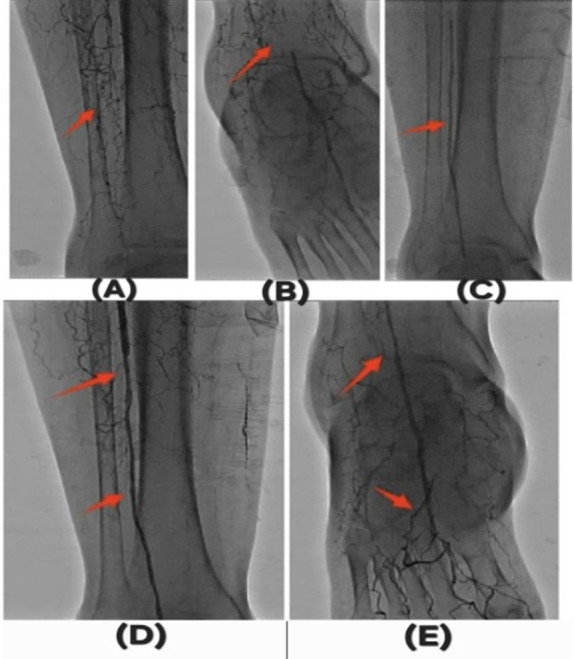
Pre- and post-angioplasty angiographic findings. (a-b) Preoperative digital subtraction angiography demonstrates infrapopliteal arterial occlusions involving both anterior and posterior tibial arteries. (c) Recanalization achieved using a 2.0 mm drug-coated balloon (DCB). (d-e) Postoperative images show successful revascularization and restoration of distal runoff.

Endovascular revascularization was successfully performed in all patients using standard percutaneous techniques. Balloon angioplasty constituted the primary revascularization strategy, with stent implantation required in only 6.3% of cases due to flow-limiting dissection or residual stenosis. Surgical conversion was necessary in 6.3% of patients. Representative angiographic images demonstrating preprocedural occlusion, intraprocedural recanalization, and restoration of distal runoff are shown in Fig.1 (A-E).

### Hemodynamic Outcomes:

Hemodynamic assessment revealed a marked improvement in lower-limb perfusion following revascularization. The mean ankle-brachial index (ABI) increased significantly from 0.61 ± 0.20 preoperatively to 0.89 ± 0.23 postoperatively (paired t-test, p < 0.001), indicating effective restoration of arterial flow (Table-II).

**Table-II:** Procedural Characteristics and Clinical Outcomes.

Variable	n / Mean ± SD	% / Range
ABI (preoperative)	0.61 ± 0.20	0.20-0.90
ABI (postoperative)	0.89 ± 0.23	0.20-1.20
Mortality	1	1.3
Primary patency	53	67.1
Secondary patency	22	27.8
Amputation-free survival	71	89.9
Stent implantation	5	6.3
Major amputation	3	3.8
Minor amputation	7	8.9
Surgical conversion	5	6.3

***Abbreviations:*** ABI, ankle-brachial index.

### Clinical Outcomes and Limb Salvage:

**Fig.2:**
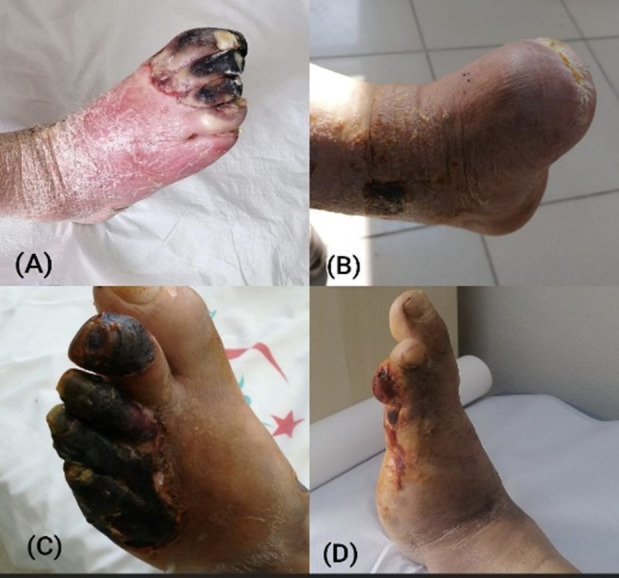
Representative clinical examples illustrating revascularization and limb salvage. (a) Preoperative photo with ischemic ulceration. (b) Postoperative photo with major amputation due to non-salvageable tissue. (c) Preoperative photo showing forefoot gangrene. (d) Postoperative photo after major amputation following failed limb salvage.

During follow-up, limb preservation was achieved in the majority of patients. Amputation-free survival was observed in 89.9% of the cohort. Major amputations occurred in 3.8% of patients, whereas minor amputations were required in 8.9%, primarily due to irreversible tissue necrosis despite successful revascularization. Overall mortality during follow-up was low (1.3%); however, detailed classification of causes of death was not consistently available. Representative clinical images illustrating ischemic tissue loss before intervention and limb outcomes following revascularization are presented in Fig.2 (A-D).

### Patency and Survival Analysis:

**Fig.3:**
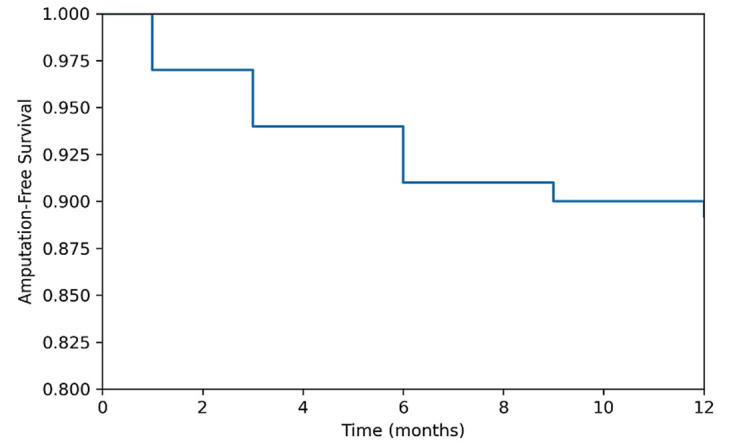
Kaplan-Meier curve demonstrating 12-month amputation-free survival following endovascular revascularization in patients with chronic limb-threatening ischemia.

Primary patency was maintained in 67.1% of patients, while secondary patency was achieved in an additional 27.8% following reintervention. Kaplan-Meier analysis demonstrated a 12-month amputation-free survival rate of 89.2% (95% CI, 82.1-96.3). The Kaplan-Meier curve illustrating amputation-free survival over time is shown in Fig.3.

## DISCUSSION

This investigation proves that endovascular revascularization within a structured multidisciplinary limb salvage program is associated with good clinical and hemodynamic outcomes for patients with advanced chronic limb-threatening ischemia. Even though there was a high incidence of severe ischemia and anatomical disease, there were good amputation-free survival rates and low major amputation rates for this high-risk group of patients. This investigation is consistent with contemporary research validating revascularization and good limb salvage outcomes for CLTI.^9^-^13^

Recent studies have demonstrated that endovascular interventions can produce limb salvage rates comparable to surgical bypass grafting, especially for patients with high operative risk and complex comorbidity.^14^,^15^ Consistent with this evidence, this study’s results indicate that an endovascular-first strategy can be safely and effectively implemented for CLTI patients. However, effective management of CLTI requires not only technical revascularization but also other aspects. The multidisciplinary approach used in this study, including endovascular therapy, medical treatment, and rehabilitation, likely played a crucial role in improving clinical outcomes. Previous reports have demonstrated that multidisciplinary limb salvage therapy can effectively reduce the rates of major amputations and improve AFS, especially for patients with advanced disease stages.^10^,^16^

The hemodynamic result of revascularization was significant, as evidenced by the significant improvement in ankle brachial index (ABI). Restoration of blood supply remains a significant factor for healing of wounds and preservation of limbs, despite the lack of vessel patency over long periods of time.^17^,^18^ In our series, primary patency rates were moderate, which was expected, given the extent of disease, including total occlusions. However, secondary patency rates and limb salvage were high, suggesting that revascularization of limbs may be more relevant to CLTI than vessel patency.^17^,^18^ The Kaplan-Meier analysis of our series showed a favorable rate of AFS at 12 months, consistent with contemporary series of CLTI patients.^10^,^17^ Although AFS, by definition, reflects all-cause mortality, lack of detailed mortality data, including cardiovascular and non-cardiovascular mortality, must be considered when evaluating this composite outcome measure. In addition, diabetes mellitus and total arterial occlusion were significant predictors of reduced AFS, emphasizing the need for surveillance of individual risk groups.^9^,^19^ Another important finding of this study is the low prevalence of stent implantation. The implantation of stents was restricted to cases with flow-limiting dissection or residual stenosis. This vessel-preserving strategy could help avoid complications related to the implantation of stents, particularly in the distal segments of the arteries. This strategy is consistent with current trends toward minimal metal implantation and optimal anti-thrombotic therapy for patients with PAD.^12^,^20^

### Strengths of this study:

It includes the homogeneity of the patient cohort with advanced CLTI, mostly characterized by severe ischemia and complex disease. All patients underwent a well-structured and multidisciplinary limb salvage program with standardized medical therapy, wound care, and follow-up. This increases the consistency and applicability of this study. The clinically relevant endpoints used in this study, such as amputation-free survival, combined with objective hemodynamic measurements with ABI, increase the validity of this study.

### Limitations:

The retrospective, single-center approach may limit generalization, and there is a risk of selection bias. The lack of a control group prevents direct comparison of our approach with surgical or conservative management strategies. Furthermore, the relatively short follow-up may not allow for evaluation of long-term durability, and multivariable-adjusted analyses were not possible because of limited events. Finally, no assessment of wound healing or quality of life was made. Future research should be directed toward prospective multicenter studies with larger cohorts to validate our findings and expand generalization. Comparative analyses of endovascular and surgical management strategies, along with multivariable-adjusted models, need to be conducted to fully understand optimal management strategies. Finally, assessment of patient-reported outcomes, including wound healing, functional status, and quality of life, along with long-term follow-up, is necessary to fully understand the effect of multidisciplinary endovascular management strategies in CLTI.

### Limitations:

This study has several limitations that should be acknowledged. First, its retrospective and single-center design may limit generalizability and introduces the potential for selection bias. Second, the absence of a surgical or conservatively treated control group precludes direct comparative conclusions regarding treatment strategies. Third, although follow-up was standardized, the overall duration was relatively limited, and longer-term outcomes beyond 12 months could not be fully assessed. Additionally, multivariable-adjusted analyses were not performed due to the sample size and number of events, which may limit the ability to identify independent predictors of adverse outcomes. Finally, wound healing and quality-of-life measures were not systematically quantified, representing areas for future prospective investigation.

## CONCLUSION

Endovascular revascularization performed within a structured multidisciplinary limb-salvage program is a safe and effective treatment strategy for patients with advanced chronic limb-threatening ischemia. Despite severe clinical presentation and complex arterial anatomy, this approach achieves significant hemodynamic improvement, high amputation-free survival, and low rates of major limb loss. These findings highlight the critical role of coordinated multidisciplinary care combined with contemporary endovascular techniques and optimal medical therapy in improving limb preservation outcomes in high-risk CLTI patients.
